# Effects of a 24-week resistance exercise program on Alzheimer’s disease brain signatures in cognitively unimpaired older adults: a secondary analysis of the AGUEDA randomized controlled trial

**DOI:** 10.1093/ageing/afag086

**Published:** 2026-04-12

**Authors:** Javier Sanchez-Martinez, Patricio Solis-Urra, Beatriz Fernandez-Gamez, Javier Fernández-Ortega, Lucía Sánchez-Aranda, Kirk I Erickson, Francisco B Ortega, Irene Esteban-Cornejo

**Affiliations:** Department of Physical Education and Sports, Faculty of Sport Sciences, Sport and Health University Research Institute (iMUDS), University of Granada, Granada, Spain; Department of Physical Education and Sports, Faculty of Sport Sciences, Sport and Health University Research Institute (iMUDS), University of Granada, Granada, Spain; Neuroscience Institute Orlando, AdventHealth Research Institute, Orlando, FL, USA; Faculty of Education and Social Sciences, Andrés Bello University, Viña del Mar, Valparaíso, Chile; Department of Physical Education and Sports, Faculty of Sport Sciences, Sport and Health University Research Institute (iMUDS), University of Granada, Granada, Spain; Department of Physical Education and Sports, Faculty of Sport Sciences, Sport and Health University Research Institute (iMUDS), University of Granada, Granada, Spain; Department of Physical Education and Sports, Faculty of Sport Sciences, Sport and Health University Research Institute (iMUDS), University of Granada, Granada, Spain; Neuroscience Institute Orlando, AdventHealth Research Institute, Orlando, FL, USA; Department of Psychology, University of Pittsburgh, Pittsburgh, PA, USA; Department of Physical Education and Sports, Faculty of Sport Sciences, Sport and Health University Research Institute (iMUDS), University of Granada, Granada, Spain; Centro de Investigación Biomédica en Red Fisiopatología de la Obesidad y Nutrición (CIBEROBN), Instituto de Salud Carlos III, Madrid, Spain; Faculty of Sport and Health Sciences, University of Jyväskylä, Jyväskylä, Finland; Department of Physical Education and Sports, Faculty of Sport Sciences, Sport and Health University Research Institute (iMUDS), University of Granada, Granada, Spain; Centro de Investigación Biomédica en Red Fisiopatología de la Obesidad y Nutrición (CIBEROBN), Instituto de Salud Carlos III, Madrid, Spain; Instituto de Investigación Biosanitaria (ibs.GRANADA), Granada, Spain

**Keywords:** aging, brain cortical thickness, cognition, diffusion magnetic resonance imaging, exercise, older people

## Abstract

**Background:**

Brain imaging markers may help detect early cognitive decline and Alzheimer’s disease (AD). Although exercise-related effects on AD-specific brain signatures remain unclear.

**Objective:**

To examine the effects of a 24-week resistance exercise (RE) program on AD brain signatures in cognitively unimpaired older adults, to explore potential moderators and to assess associations with cognition, including mediation effects.

**Methods:**

This secondary analysis of a single-site, two-arm, single-blinded randomized controlled trial included 90 participants (72 ± 4 years; 58% female) randomly assigned by a blind external researcher to an RE group (3 sessions/week, 60 min/session, n = 46) or a wait-list control group (CG, n = 44). T1- and diffusion-weighted MRI were acquired at baseline and post-intervention. Primary outcomes were thickness/volume and grey matter mean diffusivity (GMMD) signatures, derived from cortical and hippocampal regions. Moderators included age, sex, education, multimorbidity, apolipoprotein E ϵ4 status, amyloid beta (Aβ) status and baseline AD brain signatures. Secondary outcomes included cognitive function. Outcome measures and analyses were conducted by staff blinded to intervention assignment.

**Results:**

Compared with the CG, the RE group showed a reduction in the thickness/volume signature (−0.23 standardized mean difference [SMD]; 95% CI, −0.43 to −0.02), but no effect on the GMMD signature (0.08 SMD; 95% CI, −0.13 to 0.29). Aβ-status moderated the effect, as Aβ-positive participants in the RE group showed a larger reduction in the thickness/volume signature than those in the CG (−0.64 SMD; 95% CI, −1.09 to −0.18), whereas no effect was observed in Aβ-negative participants. Thickness/volume and GMMD reductions were associated with improvements in executive function and attentional/inhibitory control, respectively. Changes in AD signatures did not mediate cognitive outcomes.

**Conclusion:**

Our findings suggest that reductions in the macrostructural AD signature following a 24-week RE program may reflect adaptive, rather than detrimental, brain changes, particularly in Aβ-positive older adults, as these changes were associated with improved executive function.

**Trial registration:**

Registered on Clinicaltrials.gov (Identifier: NCT05186090).

## Key Points

- Resistance exercise reduced the thickness/volume signature in AD-related brain regions in cognitively unimpaired older adults, with stronger effects in amyloid beta-positive participants; however no impact was found on grey matter mean diffusivity signature.
- The effects were consistent across alternative methods used to compute Alzheimer’s disease brain signatures, including cortical thickness or volume.
- Reductions in the thickness/volume signature were associated with improvements in executive function.
- The findings support the feasibility of resistance exercise as a low-cost, scalable strategy to potentially prevent cognitive decline; however, longer follow-up is required to determine whether the observed brain changes are associated with a meaningful delay in long-term AD-related cognitive decline.

## Introduction

Brain imaging markers hold promise for the early detection of cognitive impairment, and conversion to diagnosed dementia [1]. Composite scores derived from structural MRI metrics in Alzheimer’s disease (AD) vulnerable regions, commonly referred to as ‘AD brain signatures’, have been shown to predict dementia risk. In particular, lower AD signature based on cortical thickness—a key predictor of AD progression [2]—is linked to a higher risk of dementia in both cognitively unimpaired adults and individuals with mild cognitive impairment [2–4]. Reduced cortical thickness has been observed in amyloid beta (Aβ) positive individuals, independent of apolipoprotein E ϵ4 (APOE4) status [5]. In addition to macrostructural changes, higher grey matter mean diffusivity (GMMD) in AD-related regions, reflecting microstructural disruption, has been associated with increased AD risk [3]. Longitudinal evidence further suggests greater increases in cortical mean diffusivity in early stages of AD, particularly among Aβ-positive individuals [6]. Together, these findings highlight the predictive value of AD brain signatures. However, emerging evidence indicates that changes in AD brain signatures may not be uniform across the AD continuum. In particular, non-linear or biphasic trajectories of macrostructural and microstructural brain changes have been proposed in cognitively unimpaired individuals at early stages of AD. [7] In this context, identifying whether interventions can modify AD brain signatures is highly relevant for preventing cognitive decline and reducing AD vulnerability.

Accumulating evidence suggests that exercise influences brain structures related to AD among cognitively unimpaired older adults; however, studies examining the effects of exercise on composite AD brain signatures remain scarce. A 2-year multimodal intervention that included exercise reported no effect on AD-signature cortical thickness in older adults at increased risk for dementia [8]. Aerobic exercise studies have shown volume increases in frontal and temporal regions, [9] and the hippocampus, [10, 11] although several trials have reported null effects on hippocampal volume [12–14]. To date, no randomized controlled trial (RCT) has examined the effects of exercise on GMMD in AD-related regions. A previous 3-month supervised walking quasi-experimental study reported increased in GMMD in cognitively unimpaired older adults, interpreted as microstructural remodelling; however, the absence of a control group precludes attributing these changes specifically to exercise [15].

Resistance exercise (RE) has emerged as a potential alternative to aerobic exercise, particularly in older adults, with evidence suggesting structural brain effects in regions vulnerable to AD that may reduce disease risk or slow progression [16]. Six months of RE increased posterior cingulate cortex, whereas no effects were observed on hippocampal volume. [17] Another study reported null effects on hippocampal volume after 52 weeks of once- or twice-weekly RE [18]. Given the potential role of RE in preventing or delaying the onset of dementia and AD, [16, 19] and the heterogeneity of prior regional findings, further investigation into its effects on AD-related macrostructural and microstructural brain changes using composite AD brain signatures is warranted.

Accordingly, the primary aim of this study was to investigate the effects of an RE program on AD brain signatures (thickness/volume and GMMD signatures) in cognitively unimpaired older adults. Secondary aims were to identify potential moderating factors and to assess associations between changes in AD brain signatures and cognitive outcomes, including the potential mediating role of AD brain signatures in the effects of RE on cognition. We hypothesized that: (i) the RE program would increase thickness/volume and reduce GMMD signatures; (ii) changes in AD brain signatures would be associated with improvements in cognitive function; and (iii) AD brain signatures would mediate the effect of RE group allocation on cognitive outcomes.

## Methods

### Study design and participants

Our study was conducted under the framework of the AGUEDA trial (‘Active Gains in brain Using Exercise During Aging’) [20]., registered on Clinicaltrials.gov (Identifier: NCT05186090; https://clinicaltrials.gov/study/NCT05186090), including a prespecified statistical analysis plan. AGUEDA is a single-site, 2-arm, single-blind RCT. Participants were recruited from community-dwelling older adults in the city of Granada (Spain) and surrounding areas from March 2021 to May 2022. Ninety cognitively unimpaired older adults (65–80 y) were randomly assigned to a 24-week RE group (n = 46) or a wait-list control group (CG, n = 44). The study followed the principles of the Declaration of Helsinki and was approved by the Research Ethics Board of the Andalusian Health Service (CEIM/CEI Provincial de Granada; #2317-N-19). All participants provided written informed consent once all study details were explained. Protocols are deposited at GitHub (https://github.com/aguedaprojectugr). The results are reported in accordance with the Consolidated Standards of Reporting Trials statement (Supplementary material: Appendix S1) [21]. A preprint of this manuscript has been uploaded to medRxiv [22]. All outcome measures and analyses were conducted by staff blinded to intervention assignment. Further methodological details, including statistical analysis plan, intervention and comparator delivery and adverse events, have been previously described [20, 23].

### Eligibility criteria

Details of the inclusion and exclusion criteria have been published previously in the study protocol [20]. Briefly, eligibility criteria were as follows: (i) older adults aged 65–80 years; (ii) physically inactive, defined as not having participated in any RE program in the previous 6 months or accumulating <600 METs-min/week on the International Physical Activity Questionnaire; [24] (iii) cognitively unimpaired, defined by scores of ≥26 points on the Spanish version of the modified Telephone Interview of Cognitive Status (STICS-m), [25] ≥25/30 on the Mini-Mental State Examination (MMSE), [26] and on the Montreal Cognitive Assessment (MoCA) according to age (<71 years: ≥24/30; 71–75 years: ≥22/30; >75 years: ≥21/30); [27] and (iv) absence of significant depressive symptoms at baseline, defined as score < 15 on the Geriatric Depression Scale [28].

### Randomization

Participants were randomized by a blinded external researcher using a 1:1 allocation ratio, stratified by age (<72 or ≥ 72 years) and sex (male or female), to the RE or CG group. Details of the randomization procedure is provided in the trial protocol [20].

### Intervention

A detailed description of the RE intervention, following the Consensus on Exercise Reporting Template (CERT), has been previously published [29]. Participants in the RE group attended 60-min supervised sessions three times per week for 24 weeks at the Sport and Health University Research Institute, University of Granada (Spain), led by professional trainers with a bachelor’s degree in Sport and Exercise Sciences. Training was conducted in groups of 4–6 participants. Each sessions comprised an 8-min warm-up, a 45-min main component and a 7-min cool-down. The main component included a combination of upper and lower limb exercises using elastic bands and body weight (three sets of eight exercises), targeting an intensity ranging from four to eight out of 10 of Borg’s Rating of Perceived Exertion scale, [30] depending on the prescribed intensity level for each week [29]. Exercise volume and intensity were individualized and progressed based on elastic band resistance (TheraBand; seven colour-coded levels), repetitions, motor complexity (three levels, 8 weeks each), number of sets and rest intervals (three sets, 60-s rest), execution time (40–60 s) and movement velocity (as fast as possible). Participants in the RE and wait-list CG were instructed to maintain their usual lifestyle; those in the wait-list CG were offered the exercise program after completing post-intervention assessments.

### Attendance

Session attendance was calculated as the proportion of completed RE sessions relative to the 72 prescribed sessions. Missed sessions were recorded and rescheduled to maximize participation. A minimum of 80% of attendance was considered as meeting the RE protocol, and those above that threshold were further excluded in the per-protocol analysis [20].

### Image acquisition

Magnetic resonance imaging (MRI) was performed on a 3 T scanner (Siemens Magnetom PRISMA Fit) equipped with a 64-channel head coil at the Mind, Brain and Behaviour Research Centre (CIMCYC), University of Granada. T1-weighted magnetization-prepared rapid acquisition gradient echo (MPRAGE) sequences were acquired for structural brain scans (parameters: sagittal, 0.8 mm isotropic resolution, echo/inversion/repetition times = 2.31/1060/2400 ms, field of view = 256 mm, 224 slices; acquisition time: 6 min 38 seconds). Diffusion-weighted images (DWI) were acquired using a diffusion sequence (parameters: Resolution: 2 × 2 × 2 mm, TE/TR = 95.6/2800 ms, multiband factor = 4, b-values of 1500, 3000 s/mm^2^, 64 gradient directions, acquisition time: 6 min 18 seconds).

### T1-weighted MPRAGE MRI processing and measurements

#### Image pre-processing

T1-weighted MPRAGE images were processed using FreeSurfer 7.4.1 (https://surfer.nmr.mgh.harvard.edu) on Neurodesk, [31] with the following three steps for longitudinal processing [32]. The pipeline is summarized in the supplementary material (Appendix S2). First, in the cross-sectional processing, the two images (pre- and post-intervention) of each subject were independently processed using the ‘recon-all’ pipeline, with the -3 T flag (*recon-all -3 T*). Second, a within-subject template (*recon-all -base*) was created by averaging the processed images from the cross-sectional processing. Third, longitudinal processing used the outputs of the previous steps to obtain the final images (*recon-all -long*).

#### Cortical thickness and volume extraction

Cortical thickness of brain regions was extracted from FreeSurfer’s outputs files (*lh.aparc.stats* and *rh.aparc.stats* files), while subcortical volumes for the left and right hemispheres, as well as the estimated intracranial volume, were obtained from the *aseg.stats* file. All files contain the Desikan-Killiany atlas parcellation [33].

#### Image quality check

Quality control (QC) was independently performed by two researchers (JSM, JOF) following the ENIGMA Consortium Cortical QC Protocol 2.0 (https://enigma.ini.usc.edu). Image quality of the FreeSurfer parcellation output was categorized as *pass, moderate* or *fail,* following ENIGMA protocol recommendations (https://enigma.ini.usc.edu/protocols/imaging-protocols/). See Results for a summary of QC results.

### Diffusion MRI processing and measurements

#### Image pre-processing

DWI were processed on Neurodesk using command modules from MRtrix3 v3.04 [34], FSL v6.0.7.16 [35] and ANTs v2.6.0 [36]. The pipeline is summarized in the supplementary material (Appendix S3). First, the diffusion-weighted gradient scheme (bvecs/bval files) was imported during the conversion of raw NifTi images to MRtrix3 format (.mif). The preprocessing steps included denoising (*dwidenoise* command), and removal of Gibbs ringing artefacts (*mrdegibbs* command). Motion and distortion correction with FSL’s eddy and topup tools within the *dwifslpreproc* script in MRtrix3, with phase encoding set to *-pe_dir j*, and including -eddy_options (“--repol [37] --cnr_maps --slm = linear). Images acquired with a different phase encoding (n = 3) were excluded. Bias field correction was applied using the ANTs algorithm via the *dwibiascorrect* command in MRtrix3, and brain masking was done with SynthStrip (*mri_synthstrip*, v7.4.1) [38]. Diffusion tensors were computed using *dwi2tensor*, and mean diffusivity (MD) was calculated as the average of the 3 eigenvalues (λ₁, λ₂, λ₃) at each voxel. To avoid physically implausible values, negative eigenvalues were set to zero prior to computation, as described in previous studies [39, 40].

#### Grey matter mean diffusivity processing

First, the b0 image (extracted from the preprocessed DWI) was registered to its respective T1 image (T1.mgz output from FreeSurfer) using the FSL’s *epi_reg* command [41]. The resulting transformation matrix was then applied to the MD images using the *FLIRT* command. A spatial smoothing with a 6-mm full-width at half maximum Gaussian kernel was applied to the MD images using the *mrfilter* command.

To limit the analysis to grey matter voxels and minimize the potential impact of partial volume effects, individual masks for grey matter and cerebrospinal fluid (CSF) were generated. The grey matter mask was derived from each *aparc + aseg.mgz* file (FreeSurfer output) using the *5ttgen freesurfer* command in MRtrix3, which produced binary segmentations of cortical grey matter, sub-cortical grey matter, white matter and CSF. The cortical and subcortical grey matter images were extracted, merged into a single mask, and then resampled to match the resolution and voxel grid of the corresponding FreeSurfer T1 image using *antsApplyTransforms* with nearest-neighbour interpolation (*−-interpolation NearestNeighbor*) and an identity transformation *(−t identity*), ensuring no geometric transformation was applied.

As recommended [42], partial volume effects arising from CSF contamination must be addressed when analysing GMMD. A CSF mask was created from the preprocessed DWI using multi-tissue constrained spherical deconvolution in MRtrix3, executed with the *dwi2fod msmt_csd* command for multi-shell diffusion data. This approach estimated the contribution of white matter-like, grey matter-like and free water CSF-like signals within each voxel. The three tissue compartment images were normalized using *mtnormalise*. The first volume of the white matter compartment was extracted using *mrconvert*, then summed with the grey matter and CSF signals using *mrcalc*. An image reflecting the proportion of CSF signal in each voxel was generated using *mrcalc*, and the b0-to-T1 transformation matrix was applied using the *FLIRT* command. Following prior research and recommendations [15, 43], each subject’s CSF mask was binarized with a threshold of 0.5 (voxels with ≥50% CSF-like signal) with *mrcalc*. The final individual grey matter mask was generated by excluding any voxels overlapping with the CSF mask and was applied to the MD image to generate a filtered GMMD image, which contains MD values only in grey matter voxels (Supplementary material: Appendix S3).

#### Grey matter mean diffusivity extraction

Cortical and subcortical regions from the Desikan-Killiany parcellation atlas were generated for each subject using the *labelconvert* command in MRtrix3 applied to FreeSurfer’s *aparc + aseg.mgz* image. Individual masks for each region were generated with *mrcalc*. Finally, mean GMMD for each cortical and subcortical region was calculated by averaging GMMD values across all voxels within each region using *mrstats*.

#### Image quality check

Two researchers (JSM, MTRP) independently assessed raw image quality using a 4-point scale: [44] 1 = ‘excellent,’ 2 = ‘minor,’ 3 = ‘moderate’ and 4 = ‘severe’, based on motion and artefacts (e.g. spiking, ghosting, missing slices). Images rated as severe were excluded; those rated as moderate were included in the main analyses but excluded from sensitivity analyses. Additionally, automatic QC was performed using EDDY QC [45] (*−eddyqc_all* in *dwifslpreproc,* MRtrix3). Extracted metrics included average absolute motion, average relative motion, outliers’ percentage, average contrast-to-noise-ratio (CNR), and signal-to-noise-ratio (SNR) in the b0. Images exceeding thresholds (absolute motion ≥2 mm, relative motion ≥0.5 mm, outliers ≥2%, SNR ≤20, CNR ≤1.5) [46] in 2 or more metrics were excluded. See Results for a summary of QC results.

### Outcomes

#### Primary outcomes: AD brain signatures

**Figure 1:**
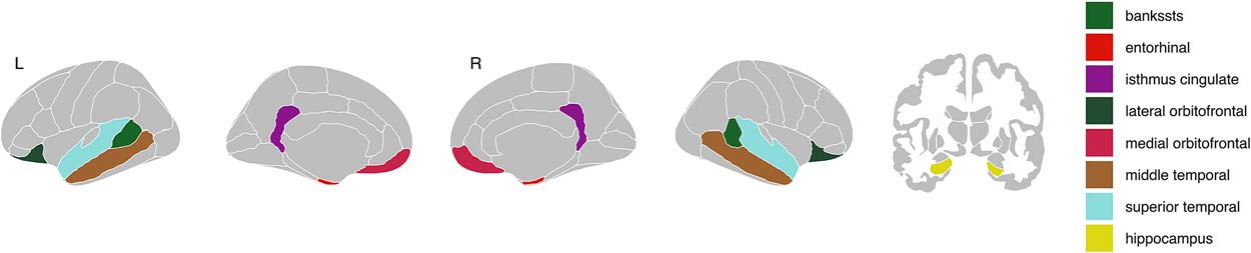
Brain regions used to compute Alzheimer’s disease brain signatures. Abbreviation: bankssts, bank of superior temporal sulcus.

AD brain signatures (i.e. thickness/volume and GMMD signatures) were computed following Williams et al. (2021) methodology [3]. The thickness/volume signature comprises a weighted average of cortical thickness in seven ROIs (entorhinal cortex, middle temporal gyrus, bank of superior temporal sulcus, superior temporal gyrus, isthmus cingulate, lateral orbitofrontal cortex and medial orbitofrontal cortex), along with hippocampal volume (Figure 1). The weights assigned to each ROI for the left and right hemispheres are detailed in Williams et al. (2021). In accordance with this methodology, structural and diffusion data for each ROI were adjusted for age, and hippocampal volume was further regressed for estimated intracranial volume to account for differences in head size. Standardized residuals for each ROI were computed separately at each time point (baseline and post assessment), then weighted and summed to calculate the thickness/volume signature scores. For the GMMD signature, the same weights used for structural data were applied to the GMMD values of each ROI, including the hippocampus.

#### Secondary outcomes: cognitive outcomes

At baseline and after the intervention, participants completed a comprehensive neuropsychological evaluation that measures global cognition (MoCA) and different cognitive domains (Supplementary material: Appendix S4). Thus, attentional/inhibitory control, episodic memory, processing speed, visuospatial processing, working memory and executive function scores were used based on a previous analysis of this sample [23].

### Statistical analysis

#### Main analysis

All statistical analyses were performed using R Statistical Software (Version 4.4.1). We used constrained longitudinal mixed models (baseline adjusted), applying the ‘LMMstar’ and ‘lme4’ packages [47, 48], with restricted maximum likelihood estimation, to test the effects of the intervention on thickness/volume and GMMD signatures. The models included time and group as a categorical fixed effect, and group-by-time interaction, with the intercept specified as a random effect. Unequal variance was allowed across time and group. Statistical significance was set at *P* < .05 without adjustments for multiple comparisons. Estimated marginal means, within-group differences and between-group differences, were calculated by ‘*emmeans’* package [49]. An intention-to-treat approach was used as the primary analysis, while per-protocol analysis (≥ 80% session attendance, >57 exercise sessions attended) as secondary/exploratory. Missing data were assumed to be missing at random and were handled within the linear mixed model analyses, as reported in the statistical analysis plan (https://clinicaltrials.gov/study/NCT05186090). Missing data were not imputed. Main intervention effects are presented as z-scores, which represent standardized mean differences (SMDs), indicating how post-exercise values deviate from baseline mean and standard deviation (SD). Effect sizes were interpreted as follows: ~0.2 SDs for a small effect, ~0.5 SDs for a medium effect and ~ 0.8 SDs for a large effect. Between-group z-score differences were computed as the exercise group minus control group. Significance was set at *P* < .05.

#### Sensitivity analysis

For the thickness/volume signature, a sensitivity analysis was conducted by excluding images with parcellation issues determined by the visual inspection (ENIGMA protocol) in the ROIs used to compute the signature. Additionally, we examined the effects of the intervention on AD signatures derived from cortical thickness and volumes, incorporating alternative methodologies that included additional brain regions (Supplementary material: Appendix S5) [50–55]. For the GMMD signature, a sensitivity analysis was performed by excluding low-quality images identified through visual QC, automated QC and those with incorrect acquisition parameters.

#### Moderator effects

Effect moderation was examined by treating age, sex (male, female), education level, multimorbidity, APOE4 carrier status, baseline Aβ burden, baseline pTau217 levels, baseline AD brain signatures as interaction terms. Age was categorized as younger (≤72 years) and older (>72 years), based on the median of the inclusion age range 65–80 years. Education level was classified as low (≤12 years of education) and high (>12 years of education). Multimorbidity was defined as two or more conditions [56], including hypertension, diabetes, cholesterol, heart disease and obesity. Participants with ≥1 APOE4 alleles were considered positive carriers. Brain Aβ deposition was assessed before and after the intervention using a PET scan with a Biograph-Vision 600 Edge Positron Emission Tomography/Computed Tomography (PET/CT) digital scanner (Siemens, Erlangen, Germany) at Virgen de las Nieves University Hospital in Granada. Aβ positivity was determined using the centiloid method, with a cut-off of >12, quantified by [18F]Forbetaben amyloid-PET scan [23, 57]. PTau-217 levels were measured using a Single molecule array (SIMOA) method on an HD-X instrument with commercial assays from Quanterix. PTau-217 were further categorized as high or low based on the median. Thickness/volume signature and GMMD signature levels were categorized as high or low based on the median. Significance was set at *P* < .05.

#### Associations of changes

Linear regression models were used to examine the associations between z-score changes in thickness/volume and GMMD signatures (predictors, analysed separately) and changes in cognitive outcomes (dependent variables). Statistical significance was set at *P* < .05.

#### Mediation analysis

We conducted a mediation analysis (‘mediation’ and ‘bruceR’ packages) [58, 59] to assess whether changes in the z-score thickness/volume or z-score GMMD signatures mediate the relationship between exercise or control conditions and cognitive outcomes. We included the outcome of interest at baseline as a covariate. We report total effects (overall impact of the predictor variable X on the outcome variable Y), direct effects (effect of X on Y without considering the mediator M) and indirect effects (mediation effect, calculated as the total effect minus the direct effect). The estimated indirect effect reflects the impact of X on Y through the mediator M. The statistical significance was assessed with percentile bootstrapping (*n* = 10.000) and 95% CIs. Outcomes are considered statistically significant when CIs do not include zero.

## Results

Participant flow from enrolment through allocation to analysis is summarized in the supplementary material (Appendix S6). Table 1 summarizes baseline characteristics of the sample. Participants had a mean age of 71.7 ± 4.0 years, 58% were females, 63% had low educational level, and mean BMI was in the overweight range. Multimorbidity was present in 68% of participants, 27% reported polypharmacy, 15% were APOE carriers and 21% were Aβ-positive. Raw values for regions of interest for cortical thickness, volumes and GMMD are described in the supplementary material (Appendix S7).

**Table 1:** Baseline characteristics for the overall sample and allocated groups

Characteristic	n	Overall	RE	CG
n		90	46	44
Age (y)	90	71.7 (4.0)	71.9 (4.2)	71.6 (3.7)
Age group	90			
Younger, ≤ 72 y		50 (56%)	26 (57%)	24 (55%)
Older, > 72 y		40 (44%)	20 (43%)	20 (45%)
Sex	90			
Male		38 (42%)	19 (41%)	19 (43%)
Female		52 (58%)	27 (59%)	25 (57%)
Education level (y)	90	11.5 (4.9)	11.2 (5.3)	11.9 (4.5)
Education level	90			
High, > 12 y		33 (37%)	14 (30%)	19 (43%)
Low, ≤ 12 y		57 (63%)	32 (70%)	25 (57%)
Body mass index (kg/m^2^)	90	28.5 (4.2)	28.3 (4.4)	28.7 (4.1)
Moderate-to-vigorous physical activity (min/day)	88	36.0 (16.9)	34.6 (16.5)	37.4 (17.5)
Functional status (SPPB), n (%)				
Best performance	90	88 (98%)	45 (98%)	43 (98%)
Middle performance		2 (2%)	1 (2%)	1 (2%)
Low performance		0 (0%)	0 (0%)	0 (0%)
Frailty, n (%)	77			
Non-frail		39 (51%)	23 (59%)	16 (42%)
Pre-frail		36 (47%)	15 (38%)	21 (55%)
Frail		2 (3%)	1 (3%)	1 (3%)
Multimorbidity, n, (%)	90			
No, 1 or no conditions		29 (32%)	15 (33%)	14 (32%)
Yes, ≥ 2 conditions		61 (68%)	31 (67%)	30 (68%)
Polypharmacy, n, (%)	90			
No medications		15 (17%)	11 (24%)	4 (9.1%)
No, 1 to 4 medications		51 (57%)	24 (52%)	27 (61%)
Yes, ≥ 5 medications		24 (27%)	11 (24%)	13 (30%)
Biomarkers				
APOE carrier	88			
Non-carrier		75 (85%)	38 (84%)	37 (86%)
Carrier		13 (15%)	7 (16%)	6 (14%)
PET Aβ (centiloid)	90	6.89 (25.16)	3.60 (23.33)	10.33 (26.78)
PET Aβ status	90			
Negative		71 (79%)	38 (83%)	33 (75%)
Positive		19 (21%)	8 (17%)	11 (25%)
pTau-217 (pg/ml)	89	−1.29 (0.45)	−1.28 (0.47)	−1.30 (0.42)
Cognitive status (score)				
Telephone Interview of Cognitive Status	90	33.91 (2.72)	33.78 (2.65)	34.05 (2.81)
Mini-Mental State Examination	90	28.92 (1.06)	29.00 (0.97)	28.84 (1.16)
Montreal Cognitive Assessment	90	25.58 (2.14)	25.28 (2.21)	25.89 (2.05)
Cognitive function (z-scores)				
Episodic memory	90	0.00 (1.01)	−0.01 (0.85)	0.01 (1.15)
Processing speed	90	−0.01 (1.00)	0.04 (0.85)	−0.06 (1.15)
Working memory	90	−0.02 (0.99)	0.01 (1.05)	−0.04 (0.94)
Attentional/inhibitory control	90	−0.01 (1.00)	0.04 (0.98)	−0.06 (1.03)
Visuospatial processing	90	−0.01 (1.00)	−0.09 (0.94)	0.08 (1.06)
Executive function	90	−0.01 (1.00)	−0.03 (1.07)	0.01 (0.94)
AD brain signatures (z-scores)				
Thickness/volume signature	90	0.00 (1.00)	0.19 (0.89)	−0.20 (1.08)
GMMD signature	86	0.00 (1.00)	−0.01 (1.07)	0.01 (0.93)

Data showed as mean (standard deviation) or n (%). Moderate-to-vigorous physical activity obtained using accelerometry following our previous approach [57]. Functional status was assessed using the Short Physical Performance Battery (SPPB), which includes three timed components: a 4-m usual pace walk, five-repetition chair stand without arm support, and a progressive standing balance test [75]. The scores were categorized as low (4–6), middle (7–9), or best performance (10–12). Frail was classified into, three stages: non-frail (score 0), pre-frail (score 1–2) and frail (score 3–5) [76], considering the five Fried frailty criteria (weight loss, exhaustion, low physical activity, slowness, weakness) [77]. Multimorbidity refers to the total number of comorbidities, including hypertension, diabetes, hypercholesterolemia, heart disease, and obesity. Polypharmacy was defined as the use of five or more medications [56]. Abbreviations: AD, Alzheimer’s disease; APOE4, apolipoprotein E ϵ4 allele; CG, wait-list control group; PET, Positron Emission Tomography; RE, resistance exercise group.

As previously reported [23], 10 of 90 participants dropped out and did not complete post-assessment measurements, including three of 46 in the RE group and seven of 44 in the control group. Mean attendance in the RE group was 85.2%; 10% of missed sessions (53/494) were rescheduled. Six participants were excluded from the per-protocol analysis because attendance was <80%; thus 87% of the 46 participants assigned to the RE group attended at least 80% of sessions. Regarding intensity, the mean achieved RPE was 5.3 per session (range: 4.7–5.8) and 4.6 per exercise (range: 3.8–5.4). Details about attendance and adverse events have been reported previously [23].

### Quality control

Details on participant inclusion and exclusion based on brain image quality are summarized in the supplementary material (Appendix S8). After applying the ENIGMA protocol, most image parcellations were rated as ‘pass’ (64.7%) or ‘moderate’ (35.3%), with none rated as ‘fail.’ Due to parcellation issues of ROIs in the ‘moderate’ category, 21 participants’ images (Pre = 11; Post = 10) were excluded from the sensitivity analysis of the thickness/volume signature. For visual DWI QC, 36.1% participant’s images were rated as ‘excellent,’ 55% as ‘minor,’ 8.3% (14 images) as ‘moderate,’ and 0.6% (1 image) as ‘severe.’ Automatic QC identified 2 low-quality images. Additionally, three images were obtained using an incorrect phase direction. Thus, five participants’ images were excluded from the main analysis (Pre = 4, Post = 1), and an additional 29 participants’ images (Pre = 11, Post = 18) were excluded from the sensitivity analysis of the GMMD signature.

### Effects on main outcomes: thickness/volume and GMMD signatures

**Figure 2:**
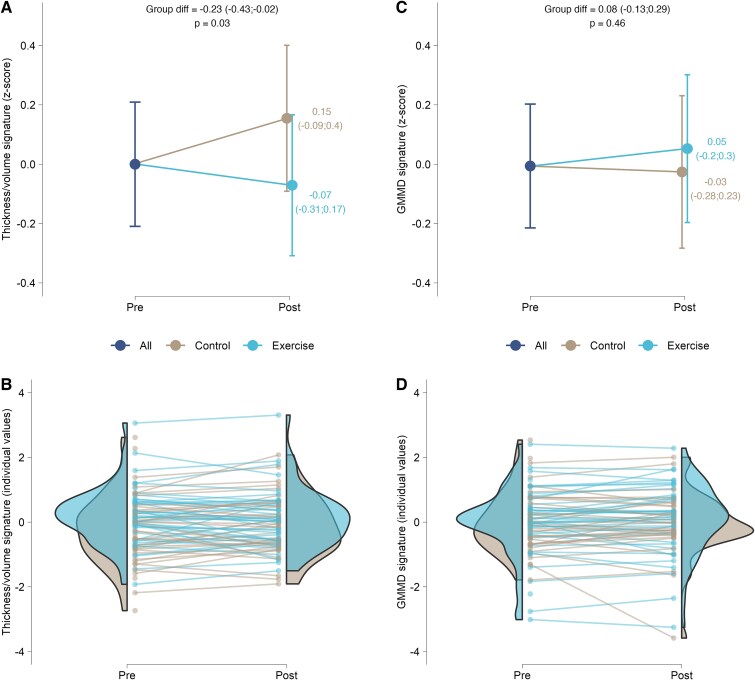
Effects of a 24-week resistance exercise program on Alzheimer’s disease brain signatures in older adults. A: Estimated marginal means at each time point (and 95% confidence intervals) for thickness/volume signature (z-score). B: Individual z-scores values at each time point for thickness/volume signature. C: Estimated marginal means at each time point (and 95% confidence intervals) for GMMD signature (z-score). D: Individual z-scores values at each time point for GMMD signature. Abbreviations: GMMD, grey matter mean diffusivity.

A significant group × time effect was found for the thickness/volume signature (Figure 2A), indicating a significant reduction in the RE group compared to the CG with a small effect size (SMD: -0.23 [95%CI, −0.43 to −0.02]; *P* = .032). The per-protocol analysis yielded a consistent result (SMD: −0.21 [95%CI, −0.42 to 0.0]) showing a trend toward significance (*P* = .055). Consistent results were found using alternative methodologies to compute the AD signature using cortical thickness or volume, and for the sensitivity analysis (Supplementary material: Appendix S9). No group × time effect was found for GMMD signature (SMD: 0.08 [95%CI, −0.13 to 0.29]; *P* = .457) (Figure 2C). Per-protocol analysis (SMD: 0.08 [95%CI, −0.13 to 0.29]; *P* = .452) and sensitivity yielded a consistent result (Supplementary material: Appendix S10).

### Moderation effects

**Figure 3:**
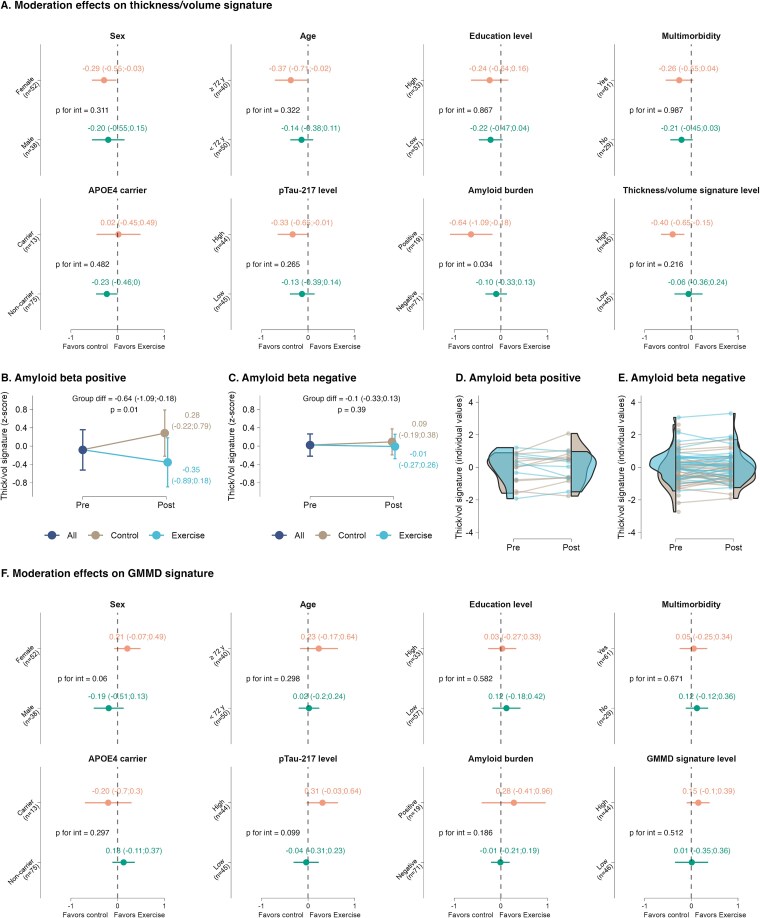
Moderator analyses of the effect of a 24-week resistance exercise program on Alzheimer’s disease brain signatures in older adults. A: Effect of moderators on thickness/volume signature, with a significance found for amyloid beta (Aβ) burden. B: Estimated marginal means at each time point (and 95% confidence intervals) for thickness/volume signature in Aβ-negative participants. C: Estimated marginal means at each time point (and 95% confidence intervals) for grey matter mean diffusivity signature in Aβ-negative participants. D: Individual z-scores values at each time point for thickness/volume signature in Aβ-positive participants. E: Individual z-scores values at each time point for thickness/volume signature in Aβ-negative participants. F: No effect of moderators on grey matter mean diffusivity signature. Abbreviations: APOE4, apolipoprotein E ϵ4, GMMD, grey matter mean diffusivity.

For the thickness/volume signature, we found a moderation effect for Aβ burden (Figure 3A) in both intention-to-treat (*P* = .034) and per-protocol analyses (*P* = .038). In Aβ-positive older adults, we found a reduction of thickness/volume signature in the RE group compared to the CG with a large effect size (SMD: -0.64 [95%CI, −1.09 to −0.18]; *P* = .010) (Figure 3B). In Aβ-negative participants, no group × time effect was found in the thickness/volume signature (SMD: -0.10 [95%CI, −0.33 to 0.13]; *P* = .394) (Figure 3C). For GMMD signature, there were no moderation effects (Figure 3F).

### Associations between changes in AD brain signatures and cognitive outcomes

**Figure 4:**
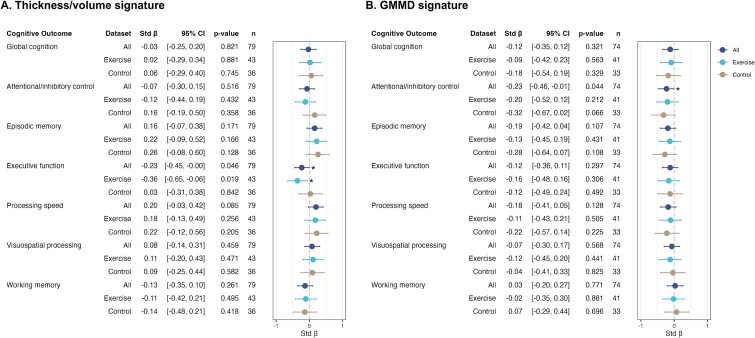
Associations between post–pre z-score differences of Alzheimer’s disease brain signatures and cognitive outcomes. A: Across all participants and in the exercise group, the thickness/volume signature was negatively associated with changes in executive function. B: Across all participants, grey matter mean diffusivity signature was negatively associated with changes in attentional/inhibitory control. Abbreviations: GMMD, grey matter mean diffusivity. ^*^  *P* < .05.

Changes in the thickness/volume signature were negatively associated with executive function, and particularly in the exercise group (Figure 4A). No significant associations were observed between changes in this signature and other cognitive outcomes. For the GMMD signature, a negative association was identified with attentional/inhibitory control (Figure 4B), while no associations were found with other cognitive outcomes.

### Testing whether AD brain signatures mediate the effects of RE on cognitive outcomes

Mediation results for the thickness/volume signature and GMMD signature on cognitive outcomes are presented in the supplementary material (Appendix S11). No mediation effects were observed for either AD brain signatures.

## Discussion

The present study aimed to examine the effects of a 24-week RE program on AD brain signatures in cognitively unimpaired older adults, as well as to explore moderating factors and association with cognitive outcomes, including mediation effects.

### Effect of the exercise intervention on AD brain signatures

Contrary to our expectations, we found that the RE intervention reduced the thickness/volume signature in cognitively unimpaired older adults. This unexpected finding may reflect biomarker-related, stage-dependent brain changes along the AD continuum, as previously described in individuals with Aβ positivity, despite the absence of cognitive impairment [7]. This finding contrasts with previous research; e.g. one study reported no effect on the cortical thickness AD signature following a 2-year multimodal intervention in older adults at increased dementia risk [8]. Similarly, a 12-month, multisite RCT in older adults with amnestic mild cognitive impairment found no effect on the cortical thickness AD signature after either moderate-to-high intensity aerobic training or lower-intensity stretching, balance and range of motion exercises, compared to a no-intervention group [60]. It is relevant to consider the differences in the methods used to compute AD signature in these studies, as well as the characteristics of the participants and populations, which could explain some of the variability in results. However, despite methodological differences, our sensitivity analyses using AD signatures based on cortical thickness and volumes further support the robustness of our results.

Research assessing the effects of exercise interventions on GMMD is scarce, and no studies have specifically examined the GMMD signature. In our study, we found no effect of the RE intervention on the GMMD signature. In contrast, one study reported increases in GMMD in the insula and cerebellum in both mild cognitive impairment and cognitively unimpaired controls after a 3-month supervised walking quasi-experimental study, suggesting improvements in neural circuits and microstructural remodelling [15]. These contrasting findings may reflect differential GMMD responses depending of the type of exercise; however, differences in study populations and outcome measures limit direct comparisons.

The observed reductions in the thickness/volume signature following RE is particularly noteworthy considering the moderating effect of Aβ burden. Specifically, this reduction was evident only in Aβ-positive participants, suggesting a differential response during early preclinical stages of AD. A biphasic trajectory of macrostructural and microstructural brain changes has been proposed in the AD continuum [7, 61], suggesting increased cortical thickness and decreased MD in AD-related regions during early preclinical AD, followed by the opposite pattern as the disease progresses and symptoms emerge. This model was supported by a study tracking longitudinal changes in thickness/volume and GMMD signatures over time [62]. Similarly, another study found reduced atrophy rates in preclinical AD stage 1 compared to stage 0, followed by increased atrophy in stages 2 and 3 [63]. Thus, the RE program may have helped to prevent early preclinical AD-related changes on thickness/volume signature in Aβ-positive participants, whereas the CG followed the expected trajectory. However, this interpretation remains speculative given the relatively short intervention period. Longer-term intervention studies with multiple assessment time points are needed to clarify the effects of RE and AD brain signatures across different stages of Aβ burden in cognitively unimpaired older adults.

Possible explanations for the biphasic trajectory of macrostructural and microstructural brain changes include biomarker alterations and neuroinflammation. In the early stages of AD, astrocytosis has been linked to increased cortical thickness and reduced MD [64], and increased levels of glial fibrillary acidic protein are associated with elevated Aβ [65]. An amyloid-triggered inflammatory response has been suggested as the cause of the increase in cortical thickness and reduction of MD during the early preclinical stage of AD stage [7]. Moreover, some studies have reported cortical thickening in AD-related regions in Aβ-positive participants compared to Aβ-negative participants during the preclinical stage of AD [66, 67]. Considering that our Aβ-positive participants were in the early preclinical AD stages, neuroinflammation and AD-related biomarker status might partially explain the observed changes in the thickness/volume signature. In this context, exercise-induced myokines released following RE [68, 69], such as interleukin-1ra or irisin, may have contributed to a reduction in neuroinflammatory activity, potentially modulation AD brain signatures. However, this interpretation remains speculative, and future studies are needed to directly investigate the role of exercise-related myokines in the interplay between neuroinflammation, AD biomarkers and AD brain signatures.

### AD brain signatures and cognition

Both macro- and microstructural brain changes have been suggested as potential mechanisms underlying cognitive changes [70]. Therefore, we explored whether changes in AD brain signatures were associated with cognitive performance.

An unexpected finding was the negative association between changes in the thickness/volume signature and executive function. Specifically, in the exercise group, reductions in the thickness/volume signature were associated with improvements in executive function. Consistent with our findings, a previous RCT reported reductions in whole brain volume accompanied by improvements in executive function after 12 months of either once-weekly or twice-weekly RE, compared to a control group (twice-weekly balance and tone training) [71]. While our findings may suggest a potential mediating role of the thickness/volume signature in the relationship between RE and executive function, no significant mediation effect was observed.

Regarding microstructural changes, we found a negative association between the GMMD signature and attentional/inhibitory control. This contrasts with a previous study that reported increases in cortical MD in response to a 3-month supervised walking quasi-experimental study, which were associated with improvements in verbal fluency and episodic memory in both individuals with mild cognitive impairment and cognitively healthy participants [15]. These discrepancies may be explained by differences in exercise modality, exercise intensity, participant characteristics, or methodological approaches.

Although no mediation of AD brain signatures on cognitive outcomes was observed in our study, exercise-related cognitive adaptations may involve mechanisms that are not fully captured by structural brain changes alone. Functional assessments, such as dual-task paradigms integrating cognition and movement, may provide complementary information to standard cognitive tests, which were not assessed in the present study, and could provide additional insight in future investigations. Previous studies have shown that resistance or multicomponent exercise in older adults improves dual-task performance and executive function [72, 73], highlighting the relevance of functional outcomes in addition to structural brain measures. Collectively, our findings emphasize the need for further research to replicate and clarify the mechanisms linking exercise, AD brain signatures and cognition.

### Clinical and practical implications

AD brain signatures derived from structural and diffusion MRI are clinically relevant because they predict future dementia risk. Lower thickness/volume signatures, reflecting macrostructural atrophy and higher GMMD signatures, reflecting microstructural disruption, have been associated with increased AD risk and disease progression [3, 62]. However, accumulating evidence indicates that these signatures may follow non-linear or biphasic trajectories across the AD continuum, particularly in early stages, when cognitively unimpaired individuals already show evidence of Aβ pathology [7, 62]. Within this framework, our findings suggest that RE may modulate the thickness/volume signature specifically in Aβ-positive, cognitively unimpaired older adults. Although no associations were observed between the thickness/volume signature and global cognition, reductions in this signature were associated with improvements in executive function. These results support the clinical relevance of the observed brain changes and suggest that exercise-related effects may extend beyond imaging markers to cognitive outcomes. Nevertheless, longer follow-up is required to elucidate whether these brain changes translate into a meaningful delay in the AD trajectory.

From a clinical perspective, these results highlight the importance of considering Aβ burden when interpreting exercise-related brain changes in older adults, as the prognostic meaning of AD brain signature changes may differ according to underlying pathology. Although the relatively small number of Aβ-positive participants warrants caution, our findings support the potential value of combining biomarker assessment with lifestyle interventions to better target individuals at higher risk of AD. The RE program used, based on elastic bands and bodyweight exercises, is low-cost, feasible and highly scalable, as reflected by high attendance rates, facilitating translation into real-world preventive strategies. In addition, we previously identified increases in strength and attentional/inhibitory control following the RE program in this sample [23], reflecting effects beyond structural brain changes and including functional outcomes that are relevant for older adults’ independence and quality of life. Larger trials with longer follow-up and greater representation of Aβ-positive individuals are needed to confirm whether such interventions can modify early brain changes along the AD continuum and ultimately reduce dementia risk.

### Strengths and limitations

A key strength of this study was the use of structural and diffusion brain imaging to assess exercise effects on AD brain signatures. Additionally, PET/CT imaging provided valuable information of brain Aβ deposition, considering the identified moderation effect of Aβ burden. Our findings remained significant even after sensitivity analyses excluded low-quality images. Given the variability in AD structural brain signature methodologies, we conducted sensitivity analyses using different approaches and found consistent results across methods. Furthermore, we explicitly detailed our structural and diffusion pipelines to enhance reproducibility. The exercise protocol was standardized and reproducible and achieved high attendance rates, slightly higher than those reported in older adults participating in exercise interventions (81%) [74], thereby supporting the internal validity of the intervention. However, the study has limitations. It focused on cognitively unimpaired older adults, so the results may not be generalizable to older adult populations with more severe cognitive decline. Additionally, only 21% of the sample was categorized as Aβ-positive; therefore, moderation effects of Aβ burden should be interpreted with caution and require further investigation. The lack of follow-up prevented assessment of potential residual effects. Moreover, no power or sample calculations were performed for the MRI-derived outcomes, as these were secondary outcomes of the trial [20]; therefore, these results should be interpreted with caution. To fully understand changes in AD brain signatures within the biphasic model, larger interventions with extended follow-up are needed to confirm the potential of exercise for delaying early structural and diffusion changes in preclinical AD. Furthermore, studies with a larger number of Aβ-positive participants are necessary to corroborate our findings.

## Conclusion

A reduction in the thickness/volume signature following a 24-week RE program may reflect adaptive brain changes linked to improvements in executive function, rather than detrimental processes, particularly in Aβ-positive cognitively unimpaired older adults. Future research should confirm these findings and explore their clinical implications for cognitive function and brain aging, to provide a more comprehensive understanding of the impact of RE.

## Supplementary Material

Supplementary_materials_afag086
