# Supplementary material for: Effects of a 24-week resistance exercise program on Alzheimer’s disease brain signatures in cognitively unimpaired older adults: a secondary analysis of the AGUEDA randomized controlled trial
Source: Age Ageing. 2026 Apr 12;55(4):afag086. doi: 10.1093/ageing/afag086 (PMC13071408; doi:10.1093/ageing/afag086)
Supplement: Supplementary_materials_afag086 [file supplementary_materials_afag086.docx]

**Supplementary material for**

**Effects of a 24-week resistance exercise program on Alzheimer’s disease brain signatures in cognitively unimpaired older adults: a secondary analysis of the AGUEDA randomized controlled trial.**

**Appendix S1.** Consolidated Standards of Reporting Trials (CONSORT) checklist.

**Appendix S2.** Longitudinal processing pipeline using FreeSurfer’s recon-all.

**Appendix S3.** Processing pipeline for regional gray matter mean diffusivity estimation.

**Appendix S4.** Global cognition, cognitive domains, and their corresponding cognitive tests.

**Appendix S5.** Brain regions used by additional methodologies to compute Alzheimer’s disease brain signatures.

**Appendix S6.** Consolidated Standards of Reporting Trials (CONSORT) flow diagram.

**Appendix S7.** Raw data for regions of interest for cortical thickness, volume, and gray matter mean diffusivity.

**Appendix S8.** Inclusion and exclusion of participants’ brain images considering image quality for main and sensitivity analyses by Alzheimer’s disease brain signature.

**Appendix S9.** Estimated marginal means in Alzheimer’s disease signature based on cortical thickness or volume using additional methodologies, and results of the sensitivity analysis excluding images with parcellation issues, stratified by amyloid beta status.

**Appendix S10.** Estimated marginal means in gray matter mean diffusivity signature, and results of the sensitivity analysis excluding low-quality images.

**Appendix S11.** Mediation analysis results.

| Appendix S1.  Consolidated Standards of Reporting Trials (CONSORT) checklist. | | | |
| --- | --- | --- | --- |
| Section/topic | No | CONSORT 2025 checklist item description | Reported on page no. |
| **Title and abstract** | | |  |
| Title and structured abstract | 1a | Identification as a randomised trial | 1 |
|  | 1b | Structured summary of the trial design, methods, results, and conclusions | 1 |
| **Open science** | | |  |
| Trial registration | 2 | Name of trial registry, identifying number (with URL) and date of registration | 3 |
| Protocol and statistical analysis plan | 3 | Where the trial protocol and statistical analysis plan can be accessed | 3, Solis et al., 2023 |
| Data sharing | 4 | Where and how the individual de-identified participant data (including data dictionary), statistical code and any other materials can be accessed | 3, Solis et al., 2023 |
| Funding and conflicts of interest | 5a | Sources of funding and other support (eg, supply of drugs), and role of funders in the design, conduct, analysis and reporting of the trial | 12 |
|  | 5b | Financial and other conflicts of interest of the manuscript authors | 12 |
| **Introduction** | | |  |
| Background and rationale | 6 | Scientific background and rationale | 2 |
| Objectives | 7 | Specific objectives related to benefits and harms | 2, 3 |
| **Methods** | | |  |
| Patient and public involvement | 8 | Details of patient or public involvement in the design, conduct and reporting of the trial | Solis et al., 2023 |
| Trial design | 9 | Description of trial design including type of trial (eg, parallel group, crossover), allocation ratio, and framework (eg, superiority, equivalence, non-inferiority, exploratory) | 3, Solis et al., 2023 |
| Changes to trial protocol | 10 | Important changes to the trial after it commenced including any outcomes or analyses that were not prespecified, with reason | NA |
| Trial setting | 11 | Settings (eg, community, hospital) and locations (eg, countries, sites) where the trial was conducted | 3 |
| Eligibility criteria | 12a | Eligibility criteria for participants | 3, Solis et al., 2023 |
|  | 12b | If applicable, eligibility criteria for sites and for individuals delivering the interventions (eg, surgeons, physiotherapists) | NA |
| Intervention and comparator | 13 | Intervention and comparator with sufficient details to allow replication. If relevant, where additional materials describing the intervention and comparator (eg, intervention manual) can be accessed | 3, Fernandez-Gamez et al., 2023 |
| Outcomes | 14 | Prespecified primary and secondary outcomes, including the specific measurement variable (eg, systolic blood pressure), analysis metric (eg, change from baseline, final value, time to event), method of aggregation (eg, median, proportion), and time point for each outcome | 5 |
| Harms | 15 | How harms were defined and assessed (eg, systematically, non-systematically) | 3, Solis et al., 2023 |
| Sample size | 16a | How sample size was determined, including all assumptions supporting the sample size calculation | 13, Solis et al., 2023 |
|  | 16b | Explanation of any interim analyses and stopping guidelines | NA |
| Randomisation: |  |  |  |
| Sequence generation | 17a | Who generated the random allocation sequence and the method used | 3, Solis et al., 2023 |
|  | 17b | Type of randomisation and details of any restriction (eg, stratification, blocking and block size) | 3, Solis et al., 2023 |
| Allocation concealment mechanism | 18 | Mechanism used to implement the random allocation sequence (eg, central computer/telephone; sequentially numbered, opaque, sealed containers), describing any steps to conceal the sequence until interventions were assigned | 3, Solis et al., 2023 |
| Implementation | 19 | Whether the personnel who enrolled and those who assigned participants to the interventions had access to the random allocation sequence | 3, Solis et al., 2023 |
| Blinding | 20a | Who was blinded after assignment to interventions (eg, participants, care providers, outcome assessors, data analysts) | 3, Solis et al., 2023 |
|  | 20b | If blinded, how blinding was achieved and description of the similarity of interventions | 3, Solis et al., 2023 |
| Statistical methods | 21a | Statistical methods used to compare groups for primary and secondary outcomes, including harms | 5 |
|  | 21b | Definition of who is included in each analysis (eg, all randomised participants), and in which group | 5, Table 1, Appendix S6, Appendix S8 |
|  | 21c | How missing data were handled in the analysis | 5, Solis et al., 2023, statistical analysis plan (<https://clinicaltrials.gov/study/NCT05186090>) |
|  | 21d | Methods for any additional analyses (eg, subgroup and sensitivity analyses), distinguishing prespecified from post hoc | 5, 6 |
| **Results** | | |  |
| Participant flow, including flow diagram | 22a | For each group, the numbers of participants who were randomly assigned, received intended intervention, and were analysed for the primary outcome | 3, 6, Appendix S6 |
|  | 22b | For each group, losses and exclusions after randomisation, together with reasons | 3, Fernandez-Gamez et al., 2026, Appendix S6 |
| Recruitment | 23a | Dates defining the periods of recruitment and follow-up for outcomes of benefits and harms | 3 |
|  | 23b | If relevant, why the trial ended or was stopped | NA |
| Intervention and comparator delivery | 24a | Intervention and comparator as they were actually administered (eg, where appropriate, who delivered the intervention/comparator, how participants adhered, whether they were delivered as intended (fidelity)) | 3, 6, Fernandez-Gamez et al., 2026 |
|  | 24b | Concomitant care received during the trial for each group | 3, Fernandez-Gamez et al., 2026 |
| Baseline data | 25 | A table showing baseline demographic and clinical characteristics for each group | Table 1 |
| Numbers analysed,  outcomes and estimation | 26 | For each primary and secondary outcome, by group:  ● the number of participants included in the analysis  ● the number of participants with available data at the outcome time point  ● result for each group, and the estimated effect size and its precision (such as 95% confidence interval)  ● for binary outcomes, presentation of both absolute and relative effect size | 6, 8, Figure 2, Appendix S6, Appendix S8 |
| Harms | 27 | All harms or unintended events in each group | 6, Fernandez-Gamez et al., 2026 |
| Ancillary analyses | 28 | Any other analyses performed, including subgroup and sensitivity analyses, distinguishing pre-specified from post hoc | 8, 9, Figure 3, Figure 4, Appendix S7, Appendix S9, Appendix S10, Appendix S11 |
| **Discussion** | | |  |
| Interpretation | 29 | Interpretation consistent with results, balancing benefits and harms, and considering other relevant evidence | 10, 11, 12 |
| Limitations | 30 | Trial limitations, addressing sources of potential bias, imprecision, generalisability, and, if relevant, multiplicity of analyses | 12 |

**
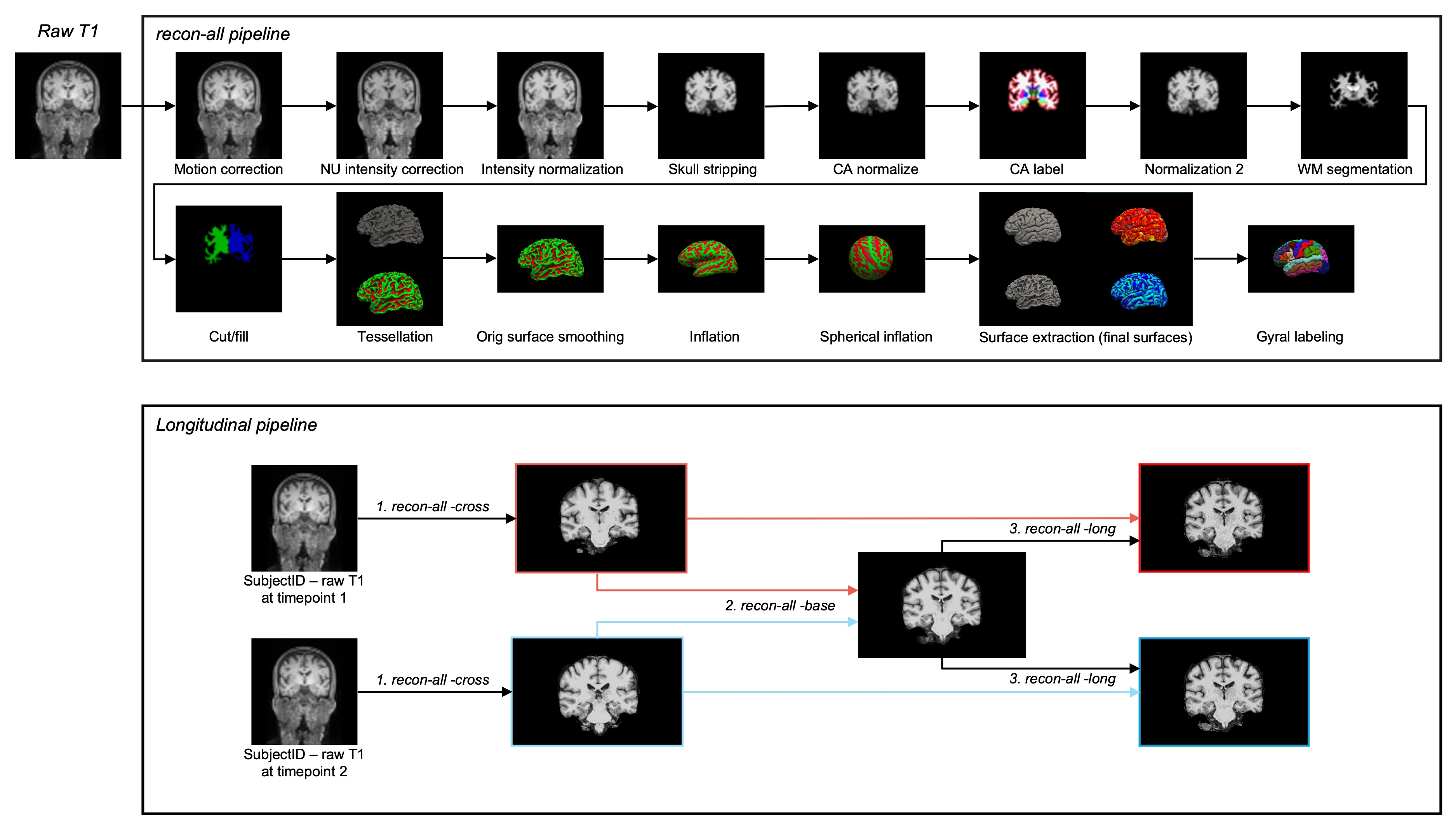
**

**Appendix S2.** Longitudinal processing pipeline using FreeSurfer’s recon-all. Abbreviation: WM, white matter.

**
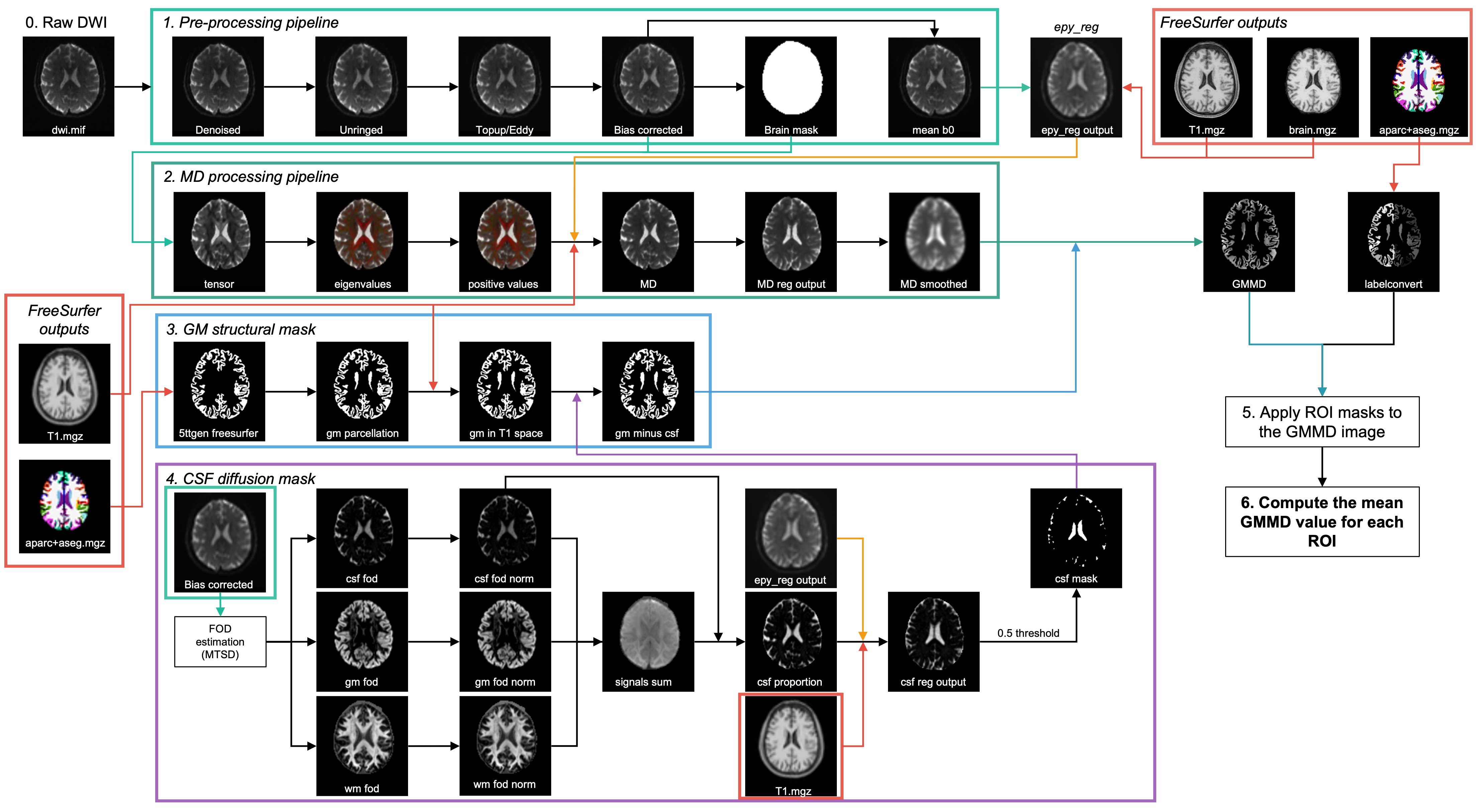
**

**Appendix S3.** Processing pipeline for regional gray matter mean diffusivity estimation. Abbreviations: csf, cerebrospinal fluid; dwi, diffusion-weighted image; gm, gray matter; GMMD, gray matter mean diffusivity; MD, mean diffusivity; ROI, region of interest; wm, white matter.

| **Appendix S4.**  Global cognition, cognitive domains, and their corresponding cognitive tests. | |
| --- | --- |
| Domain | Cognitive test |
| Global cognition | Montreal Cognitive Assessment (total score) |
| Attentional/inhibitory control | Dimensional Change Card Sort Task |
|  | Flanker |
|  | Stroop Task (incongruent trial) |
|  | Trail Making Test (part B) |
| Episodic memory | Montreal Cognitive Assessment (delayed recall) |
|  | Picture Sequence Memory Test |
|  | Rey Auditory Verbal Learning Test |
|  | Rey-Osterrieth Complex Figure Test |
| Executive function | Digit Symbol Substitution Test |
|  | Dimensional Change Card Sort Test |
|  | Trail Making Test |
|  | Spatial Working Memory Test |
| Processing speed | Digit Symbol Substitution Test |
|  | Trail Making Test (part A) |
| Visuospatial processing | Montreal Cognitive Assessment (clock drawing) |
|  | Wechsler Adult Intelligence Scale (matrix reasoning and block design) |
| Working memory | N-Back Working Memory Task |
|  | List Sorting Working Memory Test |
|  | Spatial Working Memory Task |


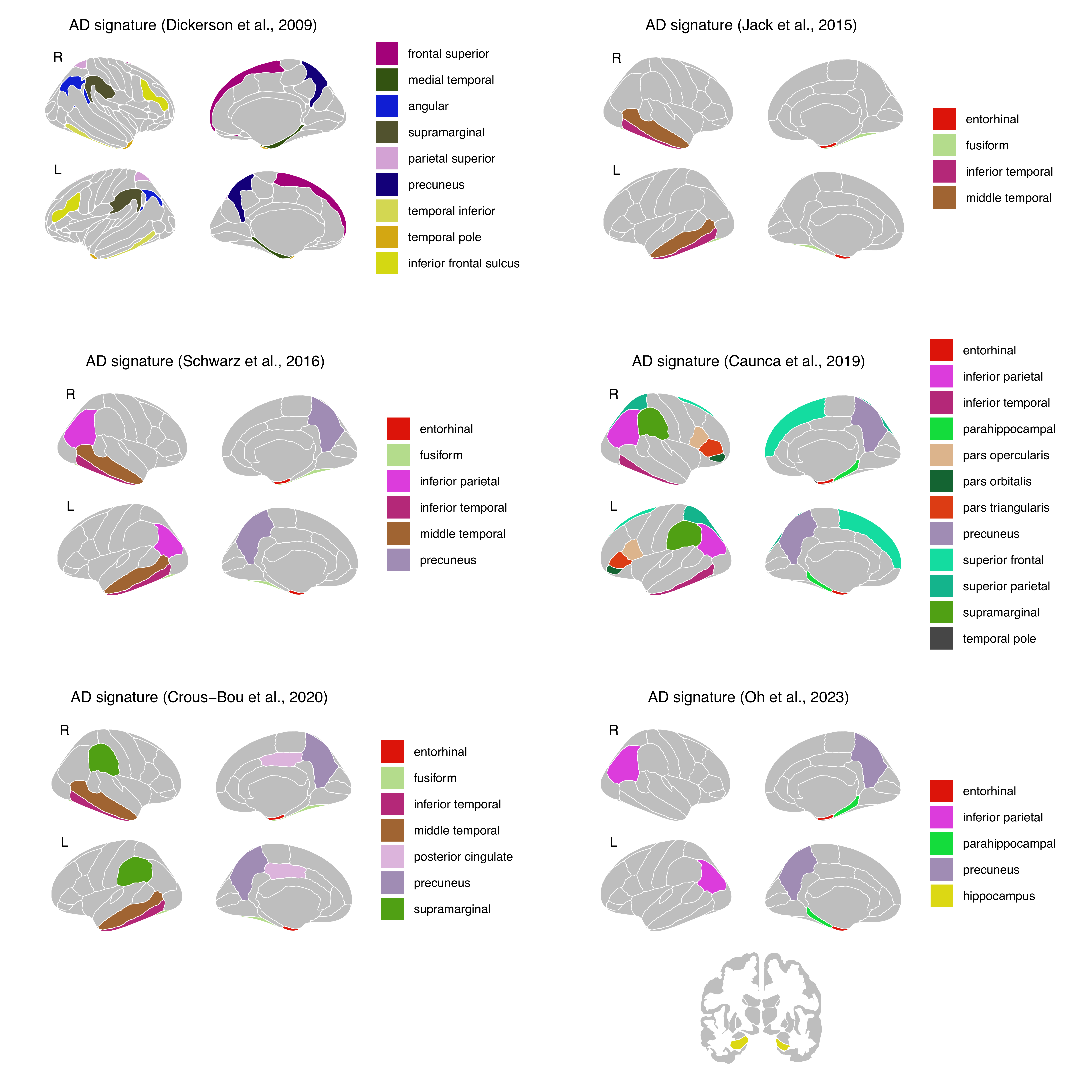


**Appendix S5.** Brain regions used by additional methodologies to compute Alzheimer’s disease brain signatures. Most Alzheimer’s disease brain signatures were derived by averaging individual cortical thicknesses across bilateral regions of interest (ROIs) and then z-scored; however, Oh et al. (2023) utilized brain volumes. ROIs for the Jack et al. (2015), Schwarz et al. (2016), Caunca et al. (2019), Crous-Bou et al. (2020), and Oh et al. (2023) approaches were obtained from FreeSurfer's outputs (‘lh.aparc.stats’ and ‘rh.aparc.stats’ files), which include the Desikan-Killiany atlas parcellation (Desikan et al., 2006). Additionally, hippocampal volumes for the Oh et al. (2023) approach were obtained from the ‘aseg.stats’ file (a FreeSurfer output), which also contains the Desikan-Killiany atlas parcellation. ROIs for the Dickerson et al. (2009) approach were obtained from FreeSurfer’s outputs (‘lh.aparc.a2009s.stats’ and ‘rh.aparc.a2009s.stats’ files), which include the Destrieux atlas parcellation (Destrieux et al., 2009). Abbreviation: AD, Alzheimer’s disease.


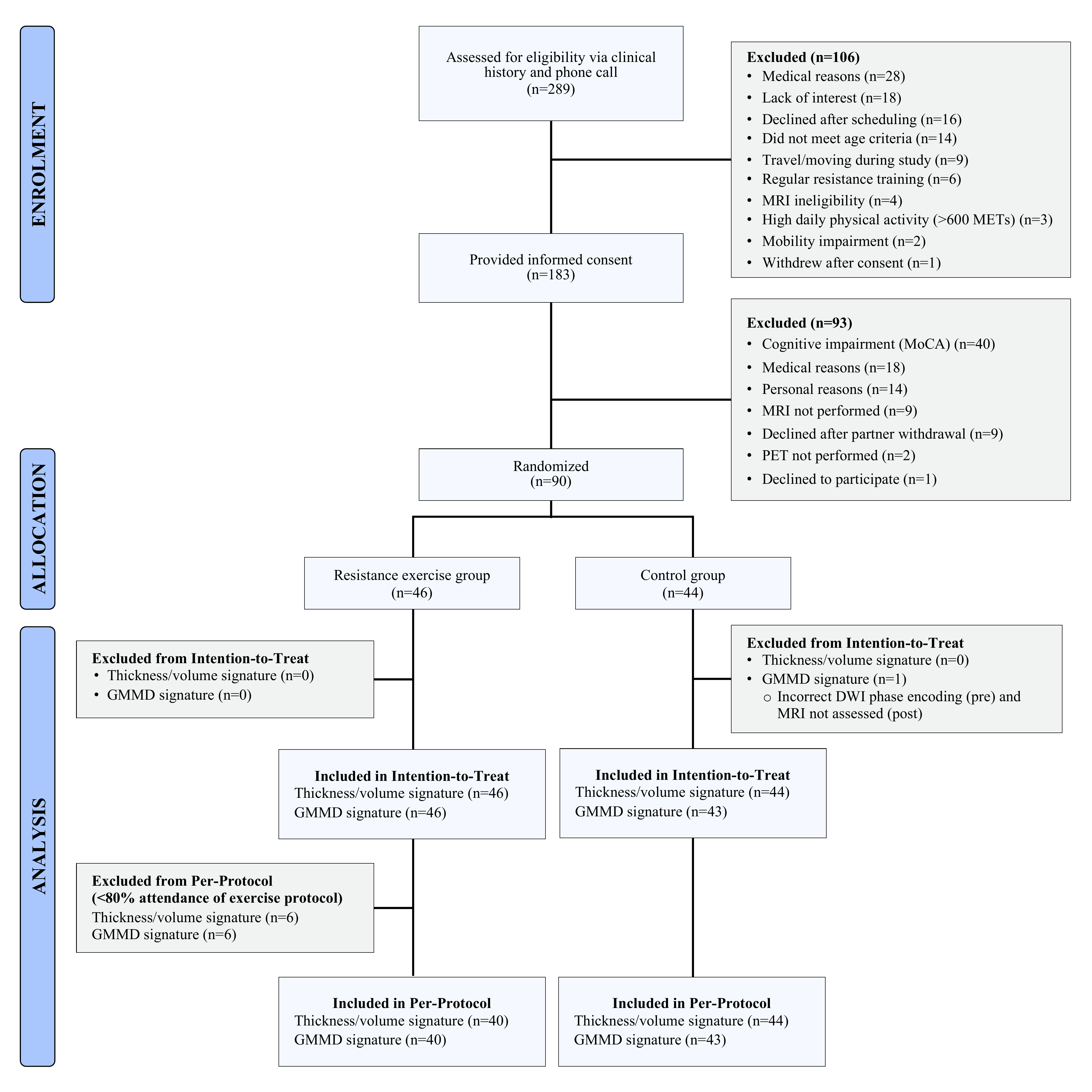


**Appendix S6.** Consolidated Standards of Reporting Trials (CONSORT) flow diagram.

| **Appendix S7.**  Raw data for regions of interest for cortical thickness, volume, and gray matter mean diffusivity. | | | | |
| --- | --- | --- | --- | --- |
| Variable | RE | | CG | |
| Cortical thickness/volume | Left | Right | Left | Right |
| Bankssts, mm | 2.45 (0.15) | 2.50 (0.14) | 2.36 (0.14) | 2.46 (0.18) |
| Entorhinal, mm | 3.11 (0.24) | 3.22 (0.19) | 3.09 (0.23) | 3.18 (0.29) |
| Isthmuscingulate, mm | 2.18 (0.16) | 2.19 (0.13) | 2.14 (0.17) | 2.15 (0.15) |
| Lateralorbitofrontal, mm | 2.60 (0.11) | 2.64 (0.14) | 2.60 (0.16) | 2.61 (0.16) |
| Medialorbitofrontal, mm | 2.45 (0.16) | 2.51 (0.14) | 2.45 (0.14) | 2.50 (0.17) |
| Middletemporal, mm | 2.73 (0.12) | 2.76 (0.12) | 2.68 (0.14) | 2.71 (0.15) |
| Superiortemporal, mm | 2.65 (0.12) | 2.68 (0.13) | 2.61 (0.19) | 2.65 (0.15) |
| Hippocampus, mm³ | 3860.57 (335.05) | 3952.33 (341.32) | 3833.07 (407.76) | 3974.68 (426.71) |
| Gray matter mean diffusivity (×10⁻³ mm²/s) |  |  |  |  |
| Bankssts | 0.61 (0.05) | 0.61 (0.04) | 0.60 (0.05) | 0.62 (0.06) |
| Entorhinal | 0.33 (0.10) | 0.34 (0.09) | 0.33 (0.08) | 0.35 (0.08) |
| Isthmuscingulate | 0.50 (0.05) | 0.49 (0.05) | 0.50 (0.06) | 0.49 (0.06) |
| Lateralorbitofrontal | 0.37 (0.09) | 0.37 (0.06) | 0.37 (0.07) | 0.37 (0.05) |
| Medialorbitofrontal | 0.26 (0.09) | 0.27 (0.08) | 0.27 (0.07) | 0.29 (0.07) |
| Middletemporal | 0.59 (0.06) | 0.59 (0.05) | 0.57 (0.05) | 0.58 (0.05) |
| Superiortemporal | 0.70 (0.09) | 0.72 (0.06) | 0.68 (0.06) | 0.70 (0.06) |
| Hippocampus | 0.43 (0.06) | 0.47 (0.06) | 0.44 (0.06) | 0.48 (0.06) |
| Data are presented as mean (standard deviation). Abbreviation: bankssts, bank of superior temporal sulcus; CG, wait-list control group; RE, resistance exercise group. | | | | |

| **Appendix S8.**  Inclusion and exclusion of participants’ brain images considering image quality for main and sensitivity analyses by Alzheimer’s disease brain signature. | | | | | |
| --- | --- | --- | --- | --- | --- |
|  | Resistance Exercise | | Control Group | | Total images |
| *For thickness/volume signature analyses* | Pre | Post | Pre | Post |  |
| All T1 images | 46 | 43 | 44 | 36 | 169 |
| **Included for main analysis** | **46** | **43** | **44** | **36** | **169** |
| Moderate QC rating with issues in parcellation of ROIs | 5 | 5 | 6 | 5 | 21 |
| **Included for sensitive analysis** | **41** | **38** | **38** | **31** | **148** |
|  |  |  |  |  |  |
| *For GMMD signature analyses* |  |  |  |  |  |
| All DWI | 46 | 42 | 44 | 36 | 168 |
| Incorrect phase direction | 1 | 0 | 2 | 0 | 3 |
| Visual DWI QC rated as severe | 0 | 0 | 0 | 1 | 1 |
| Automatic QC ≥ 2 exceeded thresholds | 0 | 0 | 1 | 1^a^ | 2 |
| **Included for main analysis** | **45** | **42** | **41** | **35** | **163** |
| Parcellation issues of ROIs in the FreeSurfer output | 4 | 5 | 3 | 5 | 17 |
| Visual DWI QC rated as moderate | 5^b^ | 6 | 0 | 2 | 13 |
| **Included for sensitive analysis** | **37** | **31** | **38** | **28** | **134** |
| Abbreviations: DWI, diffusion-weighted images; GMMD, gray matter mean diffusivity; ROIs, regions of interest; QC, quality control.  ^a^ One image corresponded to a participant image rated as severe in the visual DWI QC.  ^b^ Corresponded to a participant image with parcellation issues in the FreeSurfer output. | | | | | |

| **Appendix S9**.  Estimated marginal means in Alzheimer’s disease signature based on cortical thickness or volume using additional methodologies, and results of the sensitivity analysis excluding images with parcellation issues, stratified by amyloid beta status. | | | | | | | | | | | |
| --- | --- | --- | --- | --- | --- | --- | --- | --- | --- | --- | --- |
|  | Main analysis | | | | |  | Sensitivity analysis | | | | |
|  | **Pre** | **Post** | | |  |  | **Pre** | **Post** | | |  |
|  | **All** | **RE** | **CG** | **Group difference** | **p-value** |  | **All** | **RE** | **CG** | **Group difference** | **p-value** |
| **Overall** | n = 90 | n = 46 | n = 44 |  |  |  | n = 79 | n = 41 | n = 38 |  |  |
| Thickness/volume signature | 0 [-0.21;0.21] | -0.07 [-0.31;0.17] | 0.15 [-0.09;0.40] | -0.23 [-0.43;0.02] | **0.032** |  | 0 [-0.22;0.22] | -0.03 [-0.27;0.22] | 0.17 [-0.08;0.43] | -0.20 [-0.42;0.02] | 0.070 |
| AD signature (Dickerson et al., 2009) | 0 [-0.21;0.21] | -0.22 [-0.46;0.02] | 0.08 [-0.16;0.33] | -0.30 [-0.52;-0.09] | **0.007** |  | 0 [-0.22;0.22] | -0.23 [-0.50;0.04] | 0.06 [-0.22;0.34] | -0.29 [-0.54;-0.04] | **0.025** |
| AD signature (Jack et al., 2015) | 0 [-0.21;0.21] | -0.15 [-0.38;0.09] | 0.12 [-0.12;0.36] | -0.27 [-0.48;-0.06] | **0.012** |  | 0 [-0.22;0.22] | -0.20 [-0.46;0.06] | 0.09 [-0.19;0.36] | -0.28 [-0.53;-0.04] | **0.025** |
| AD signature (Schwarz et al., 2016) | 0 [-0.21;0.21] | -0.19 [-0.43;0.05] | 0.13 [-0.12;0.38] | -0.32 [-0.54;-0.10] | **0.005** |  | 0 [-0.22;0.22] | -0.24 [-0.50;0.03] | 0.10 [-0.18;0.37] | -0.33 [-0.59;-0.07] | **0.014** |
| AD signature (Caunca et al., 2019) | 0 [-0.21;0.21] | -0.18 [-0.42;0.05] | 0.05 [-0.19;0.29] | -0.24 [-0.44;-0.04] | **0.020** |  | 0 [-0.22;0.22] | -0.20 [-0.46;0.05] | 0.02 [-0.24;0.29] | -0.23 [-0.46;0.00] | 0.054 |
| AD signature (Crous-Bou et al., 2020) | 0 [-0.21;0.21] | -0.17 [-0.40;0.07] | 0.10 [-0.14;0.35] | -0.27 [-0.48;-0.06] | **0.011** |  | 0 [-0.22;0.22] | -0.21 [-0.46;0.05] | 0.07 [-0.19;0.34] | -0.28 [-0.52;-0.04] | **0.020** |
| AD signature^a^ (Oh et al., 2023) | 0 [-0.21;0.21] | -0.15 [-0.37;0.07] | 0.03 [-0.20;0.25] | -0.18 [-0.30;-0.05] | **0.007** |  | 0 [-0.22;0.22] | -0.16 [-0.40;0.08] | 0.01 [-0.23;0.25] | -0.17 [-0.32;-0.03] | **0.022** |
| **Aβ-negative older adults** | n = 71 | n = 38 | n = 33 |  |  |  | n = 64 | n = 35 | n = 29 |  |  |
| Thickness/volume signature | 0.02 [-0.22;0.27] | -0.01 [-0.27;0.26] | 0.09 [-0.19;0.38] | -0.10 [-0.33;0.13] | 0.394 |  | 0 [-0.26;0.26] | 0.01 [-0.27;0.29] | 0.09 [-0.21;0.39] | -0.08 [-0.33;0.17] | 0.519 |
| AD signature (Dickerson et al., 2009) | 0.06 [-0.18;0.30] | -0.11 [-0.38;0.17] | 0.05 [-0.24;0.33] | -0.15 [-0.38;0.08] | 0.192 |  | 0.03 [-0.22;0.28] | -0.13 [-0.43;0.16] | -0.02 [-0.33;0.30] | -0.12 [-0.37;0.14] | 0.378 |
| AD signature (Jack et al., 2015) | 0.01 [-0.23;0.26] | -0.10 [-0.38;0.17] | 0.09 [-0.20;0.38] | -0.20 [-0.43;0.04] | 0.099 |  | -0.01 [-0.27;0.26] | -0.15 [-0.45;0.15] | 0.05 [-0.27;0.37] | -0.20 [-0.48;0.08] | 0.157 |
| AD signature (Schwarz et al., 2016) | 0.03 [-0.21;0.27] | -0.12 [-0.40;0.16] | 0.09 [-0.20;0.38] | -0.21 [-0.45;0.03] | 0.082 |  | 0 [-0.25;0.26] | -0.16 [-0.46;0.14] | 0.04 [-0.28;0.37] | -0.21 [-0.48;0.07] | 0.144 |
| AD signature (Caunca et al., 2019) | 0.02 [-0.23;0.27] | -0.12 [-0.39;0.15] | -0.01 [-0.29;0.27] | -0.11 [-0.33;0.11] | 0.335 |  | -0.01 [-0.27;0.25] | -0.16 [-0.45;0.13] | -0.08 [-0.39;0.23] | -0.07 [-0.33;0.18] | 0.556 |
| AD signature (Crous-Bou et al., 2020) | 0.03 [-0.21;0.28] | -0.10 [-0.38;0.17] | 0.08 [-0.21;0.36] | -0.18 [-0.41;0.05] | 0.121 |  | 0 [-0.26;0.26] | -0.15 [-0.44;0.15] | 0.02 [-0.29;0.34] | -0.17 [-0.43;0.09] | 0.199 |
| AD signature^a^ (Oh et al., 2023) | -0.02 [-0.26;0.21] | -0.13 [-0.37;0.11] | -0.05 [-0.30;0.19] | -0.07 [-0.20;0.06] | 0.273 |  | -0.03 [-0.26;0.21] | -0.12 [-0.37;0.12] | -0.08 [-0.32;0.17] | -0.05 [-0.20;0.10] | 0.512 |
| **Aβ-positive older adults** | n = 9 | n = 8 | n = 11 |  |  |  | n = 15 | n = 6 | n = 9 |  |  |
| Thickness/volume signature | -0.08 [-0.52;0.36] | -0.35 [-0.89;0.18] | 0.28 [-0.22;0.79] | -0.64 [-1.09;-0.18] | **0.010** |  | 0 [-0.45;0.45] | -0.29 [-0.81;0.22] | 0.41 [-0.06;0.87] | -0.70 [-1.17;-0.23] | **0.007** |
| AD signature (Dickerson et al., 2009) | -0.23 [-0.65;0.20] | -0.72 [-1.24;-0.19] | 0.14 [-0.34;0.61] | -0.85 [-1.38;-0.33] | **0.004** |  | -0.13 [-0.65;0.40] | -0.77 [-1.43;-0.11] | 0.25 [-0.31;0.82] | -1.02 [-1.67;-0.37] | **0.006** |
| AD signature (Jack et al., 2015) | -0.06 [-0.46;0.35] | -0.38 [-0.84;0.08] | 0.20 [-0.21;0.62] | -0.59 [-1.06;-0.11] | **0.018** |  | 0.03 [-0.42;0.47] | -0.48 [-1.01;0.05] | 0.20 [-0.26;0.66] | -0.68 [-1.25;-0.11] | **0.023** |
| AD signature (Schwarz et al., 2016) | -0.11 [-0.54;0.32] | -0.54 [-1.04;-0.04] | 0.21 [-0.24;0.66] | -0.75 [-1.29;-0.22] | **0.009** |  | -0.02 [-0.54;0.50] | -0.66 [-1.29;-0.03] | 0.25 [-0.28;0.79] | -0.91 [-1.59;-0.23] | **0.013** |
| AD signature (Caunca et al., 2019) | -0.07 [-0.48;0.34] | -0.49 [-0.97;-0.01] | 0.20 [-0.24;0.65] | -0.69 [-1.14;-0.24] | **0.005** |  | 0.06 [-0.40;0.52] | -0.49 [-1.01;0.03] | 0.36 [-0.10;0.81] | -0.85 [-1.35;-0.34] | **0.004** |
| AD signature (Crous-Bou et al., 2020) | -0.12 [-0.53;0.29] | -0.45 [-0.90;0.00] | 0.16 [-0.24;0.57] | -0.62 [-1.08;-0.16] | **0.012** |  | 0 [-0.46;0.46] | -0.56 [-1.06;-0.06] | 0.23 [-0.21;0.67] | -0.79 [-1.28;-0.30] | **0.005** |
| AD signature^a^ (Oh et al., 2023) | 0.09 [-0.42;0.60] | -0.31 [-0.89;0.27] | 0.28 [-0.29;0.84] | -0.59 [-0.91;-0.26] | **0.002** |  | 0.11 [-0.56;0.78] | -0.44 [-1.20;0.32] | 0.31 [-0.44;1.05] | -0.75 [-1.10;-0.40] | **0.001** |
| Data are presented as mean change [95% confidence interval]. Significant results shown in bold (p <0.05). AD signatures were computed using cortical thickness, except for Oh et al. (2023), which utilized brain volumes. Abbreviations: Aβ, amyloid beta; AD, Alzheimer’s disease; CG, wait-list control group; RE, resistance exercise group. ^a^ Adjusted for intracranial volume. | | | | | | | | | | | |

| **Appendix S10.**  Estimated marginal means in gray matter mean diffusivity signature, and results of the sensitivity analysis excluding low-quality images. | | | | | | | | | | | |
| --- | --- | --- | --- | --- | --- | --- | --- | --- | --- | --- | --- |
|  | Main analysis | | | | |  | Sensitivity analysis | | | | |
|  | **Pre** | **Post** | | |  |  | **Pre** | **Post** | | |  |
|  | **All** | **RE** | **CG** | **Group difference** | **p-value** |  | **All** | **RE** | **CG** | **Group difference** | **p-value** |
|  | n = 89 | n = 46 | n = 43 |  |  |  | n = 77 | n = 40 | n = 37 |  |  |
| GMMD signature | -0.01 [-0.21;0.20] | 0.05 [-0.20;0.30] | -0.03 [-0.28;0.23] | -0.8 [-0.13;0.29] | 0.457 |  | -0.01 [-0.24;0.22] | 0.07 [-0.21;0.36] | 0.01 [-0.29;0.3] | 0.07 [-0.2;0.33] | 0.625 |
| Data are presented as mean change [95% confidence interval]. Abbreviations: AD, Alzheimer’s disease; CG, wait-list control group; GMMD, gray matter mean diffusivity; RE, resistance exercise group. | | | | | | | | | | | |

| **Appendix S11.**  Mediation analysis results. | | | | |
| --- | --- | --- | --- | --- |
| Mediator (change) | Outcome | Indirect Effect  (a×b) [95% CI] | Direct Effect  (c') [95% CI] | Total Effect  (c) [95% CI] |
| Thickness/volume signature | Global cognition | -0.03 [-0.10; 0.03] | 0.18 [-0.04; 0.40] | 0.15 [-0.06; 0.36] |
|  | Attentional/inhibitory control | -0.02 [-0.08; 0.03] | **0.25 [0.08; 0.44]** | **0.23 [0.09; 0.39]** |
|  | Episodic memory | -0.04 [-0.10; 0.01] | **0.20 [0.03; 0.36]** | **0.16 [0.01; 0.31]** |
|  | Executive function | 0.03 [-0.03; 0.10] | 0.05 [-0.13; 0.22] | 0.07 [-0.08; 0.22] |
|  | Processing speed | -0.03 [-0.09; 0.01] | 0.02 [-0.11; 0.16] | -0.01 [-0.13; 0.12] |
|  | Visuospatial processing | -0.04 [-0.11; 0.01] | 0.06 [-0.14; 0.26] | 0.02 [-0.17; 0.21] |
|  | Working memory | 0.01 [-0.06; 0.07] | 0.03 [-0.15; 0.23] | 0.03 [-0.14; 0.22] |
|  |  |  |  |  |
| GMMD signature | Global cognition | -0.01 [-0.05; 0.05] | 0.14 [-0.07; 0.35] | 0.13 [-0.08; 0.34] |
|  | Attentional/inhibitory control | -0.02 [-0.07; 0.05] | **0.26 [0.10; 0.42]** | **0.24 [0.09; 0.42]** |
|  | Episodic memory | -0.01 [-0.03; 0.04] | **0.17 [0.00; 0.31]** | **0.16 [0.00; 0.32]** |
|  | Executive function | -0.01 [-0.04; 0.04] | 0.11 [-0.06; 0.26] | 0.10 [-0.06; 0.26] |
|  | Processing speed | -0.01 [-0.04; 0.02] | 0.01 [-0.12; 0.14] | 0.00 [-0.12; 0.14] |
|  | Visuospatial processing | -0.01 [-0.04; 0.05] | 0.03 [-0.19; 0.23] | 0.02 [-0.18; 0.22] |
|  | Working memory | 0.00 [-0.03; 0.05] | 0.02 [-0.19; 0.22] | 0.02 [-0.17; 0.22] |
| Significant results shown in bold (when CIs do not include zero). Abbreviations: CI, confidence interval; GMMD, gray matter mean diffusivity. | | | | |
